# Asian-Pacific perspective on the psychological well-being of healthcare workers during the evolution of the COVID-19 pandemic

**DOI:** 10.1192/bjo.2020.98

**Published:** 2020-10-08

**Authors:** Nicholas W. S. Chew, Jinghao Nicholas Ngiam, Benjamin Yong-Qiang Tan, Sai-Meng Tham, Celine Yan-Shan Tan, Mingxue Jing, Renarebecca Sagayanathan, Jin Tao Chen, Lily Y. H. Wong, Aftab Ahmad, Faheem Ahmed Khan, Maznah Marmin, Fadhlina Binte Hassan, Tai Mei-Ling Sharon, Chin Han Lim, Mohamad Iqbal Bin Mohaini, Rivan Danuaji, Thang H. Nguyen, Georgios Tsivgoulis, Sotirios Tsiodras, Paraskevi C. Fragkou, Dimitra Dimopoulou, Arvind K. Sharma, Kenam Shah, Bhargesh Patel, Suktara Sharma, R. N. Komalkumar, R. V. Meenakshi, Shikha Talati, Hock Luen Teoh, Cyrus S. Ho, Roger C. Ho, Vijay K. Sharma

**Affiliations:** Department of Medicine, National University Health System, Singapore; Department of Medicine, National University Health System, Singapore; Division of Neurology, Department of Medicine, National University Health System, Singapore; and Department of Medicine, Yong Loo Lin School of Medicine, National University of Singapore, Singapore; Department of Medicine, National University Health System, Singapore; Department of Medicine, National University Health System, Singapore; Division of Neurology, Department of Medicine, National University Health System, Singapore; Division of Neurology, Department of Medicine, National University Health System, Singapore; Division of Neurology, Department of Medicine, National University Health System, Singapore; Division of Neurology, Department of Medicine, National University Health System, Singapore; Department of Neurology, Ng Teng Fong General Hospital, Singapore; Department of Intensive Care Medicine, Ng Teng Fong General Hospital, Singapore; Department of Neurology, Ng Teng Fong General Hospital, Singapore; Department of Neurology, Ng Teng Fong General Hospital, Singapore; University of Malaya, Kuala Lumpur, Malaysia; Tawau Hospital, Malaysia; Tawau Hospital, Malaysia; Dr Moewardi Hospital Surakarta, Indonesia; Cerebrovascular Disease Department, 115 People's Hospital, Vietnam; Attikon University Hospital, National and Kapodistrian University of Athens, Greece; Attikon University Hospital, National and Kapodistrian University of Athens, Greece; Attikon University Hospital, National and Kapodistrian University of Athens, Greece; Attikon University Hospital, National and Kapodistrian University of Athens, Greece; Zydus Hospital, India; Zydus Hospital, India; Zydus Hospital, India; GCS Medical College, India; Yashoda Hospital, India; Senthil Multi Specialty Hospital, India; Department of Psychiatry, Geetanjali Medical College and Hospital, India; Division of Neurology, Department of Medicine, National University Health System, Singapore; and Department of Medicine, Yong Loo Lin School of Medicine, National University of Singapore, Singapore; Department of Psychological Medicine, National University of Singapore, Singapore; Department of Psychological Medicine and Institute of Health Innovation and Technology (iHealthtech), National University of Singapore, Singapore; Division of Neurology, Department of Medicine, National University Health System, Singapore; and Department of Medicine, Yong Loo Lin School of Medicine, National University of Singapore, Singapore

**Keywords:** COVID-19, healthcare workers, psychological impact, pandemic, Asia-Pacific

## Abstract

**Background:**

The coronavirus disease 2019 (COVID-19) pandemic has led to significant strain on front-line healthcare workers.

**Aims:**

In this multicentre study, we compared the psychological outcomes during the COVID-19 pandemic in various countries in the Asia-Pacific region and identified factors associated with adverse psychological outcomes.

**Method:**

From 29 April to 4 June 2020, the study recruited healthcare workers from major healthcare institutions in five countries in the Asia-Pacific region. A self-administrated survey that collected information on prior medical conditions, presence of symptoms, and scores on the Depression Anxiety Stress Scales and the Impact of Events Scale-Revised were used. The prevalence of depression, anxiety, stress and post-traumatic stress disorder (PTSD) relating to COVID-19 was compared, and multivariable logistic regression identified independent factors associated with adverse psychological outcomes within each country.

**Results:**

A total of 1146 participants from India, Indonesia, Singapore, Malaysia and Vietnam were studied. Despite having the lowest volume of cases, Vietnam displayed the highest prevalence of PTSD. In contrast, Singapore reported the highest case volume, but had a lower prevalence of depression and anxiety. In the multivariable analysis, we found that non-medically trained personnel, the presence of physical symptoms and presence of prior medical conditions were independent predictors across the participating countries.

**Conclusions:**

This study highlights that the varied prevalence of psychological adversity among healthcare workers is independent of the burden of COVID-19 cases within each country. Early psychological interventions may be beneficial for the vulnerable groups of healthcare workers with presence of physical symptoms, prior medical conditions and those who are not medically trained.

After the initial presentation of a cluster of viral pneumonia in Wuhan, China, coronavirus disease 2019 (COVID-19) has spread rapidly and globally, with the outbreak at its peak in various countries in South Asia.^1^ Accordingly, as of the 30th June 2020, Singapore reported a total of 4846 cases per million population with 4 deaths per million population. This was followed by Malaysia (255 cases and 4 deaths per million population ); India (164 cases and 5 deaths per million population); Indonesia (105 cases and 5 deaths per million population ) and Vietnam (3 cases per million population and no reported deaths).^2^

Besides the direct health impact of COVID-19 on patients,^3^ it has placed a significant strain on healthcare workers and resources. Healthcare institutions have enforced measures such as the donning of appropriate personal protective equipment and minimising non-essential services to avoid unnecessary exposure of healthcare staff to COVID-19.^4^ It has led to considerable psychological impact on front-line healthcare staff even during the early periods of the outbreak.^5–9^ In this multicentre study on healthcare workers, we compared the psychological outcomes and its predictors during the current COVID-19 pandemic in various countries in the Asia-Pacific region.

## Method

### Study setting and population

Between the period of 29 April 2020 to 4 June 2020, healthcare workers from major tertiary healthcare institutions from India, Singapore, Malaysia, Vietnam and Indonesia were recruited. During the study period, these healthcare institutions were actively involved in the management of COVID-19 patients. The survey was extended to all healthcare workers directly involved in wards or facilities designated for managing patients with COVID-19. Study participants included medically trained (doctors and nurses) and non-medically trained personnel (administrative staff, pharmacists, cleaners, porters and technicians). All participants provided written informed consent. The survey was administered once and there were no subsequent follow-up questionnaires. The study was approved by the institutional review boards of various centres, in accordance with the principles of the Declaration of Helsinki.

### Screening questionnaire

The study questionnaire was administered in English. In all the hospitals where the survey was administered, English was the primary language of communication among the staff. The participants’ baseline information on demographic characteristics, their past medical history and symptom prevalence in the immediate previous month were obtained. Psychological outcomes were assessed using Depression Anxiety Stress Scales (DASS-21) and the Impact of Events Scale-Revised (IES-R) instruments.^10,11^

The DASS-21 is an internationally recognised screening tool used for the general population for screening depression, anxiety and stress. It is a self-administered 21-item instrument created by the University of New South Wales, Australia, which screens for depression, anxiety and stress based on the recommended severity thresholds for the depression, anxiety, stress subscales.^10^ For the purpose of this study, we examined depression, anxiety and stress with cut-off scores of >9, >7 and >14, respectively. Each DASS-21 subscales were further categorised as mild, moderate, severe and extremely severe, as follows: for depression subscales, 10–13, 14–20, 21–27, 28–42 points, respectively; for anxiety subscales, 8–9, 10–14, 15–19, 20–42 points, respectively; and for stress subscales, 15–18, 19–25, 26–33, 34–42 points, respectively.^10^

The IES-R was used to assess the extent of psychological distress among healthcare workers. This is an internationally validated 22-item screening system that has three further subcategories (intrusion, avoidance, and hyperarousal), which are associated with the symptoms displayed in post-traumatic stress disorder (PTSD).^11^ We studied PTSD specifically in relation to the COVID-19 pandemic, and did not examine other general causes of PTSD. Participants had to rate their level of distress for each component during the past 7 days on the questionnaire. The severity of psychological impact was graded from normal (0–23 points), mild (24–32 points), moderate (33–36 points), to severe (>37 points). A threshold score of ≥24 points was used to define PTSD as a clinical concern.^11^ Both DASS-21 and IES-R have been used to evaluate the psychological impact of COVID-19 in previous studies.^5,12^

### Study outcomes

The primary study outcome was the comparison of the prevalence of depression, anxiety, stress and PTSD related to COVID-19 reported by healthcare workers during the pandemic in five countries in the Asia-Pacific region. Subsequently, we explored the independent predictors of these psychological outcomes in each country.

### Statistical analyses

Continuous variables were expressed as mean and s.d., while categorical variables were expressed as absolute values (percentage). Continuous variables were compared using Student's *t*-test, while categorical variables were examined using Pearson's chi-squared test (or Fisher's exact test, where appropriate). The comparison of the prevalence of psychological outcomes among the five countries was performed using logistic regression. The stress outcome was excluded from the logistic regression model as none of the healthcare workers in Singapore, India and Malaysia were screened positive for the outcome. Multivariable logistic regression was performed for the subgroup analysis of each country to evaluate for independent associations with adverse psychological outcome and traditional covariates. A *P*-value of less than 0.05 was deemed significant for this study. All statistical analyses were performed using IBM SPSS Statistics for Windows, Version 25.0.

## Results

### Participants

Of the 1300 healthcare workers invited to participate in this study, 1146 (88.2% response rates) agreed to participate in the study. There were 384 (33.5%) respondents from India, 277 (24.2%) from Singapore, 250 (21.8%) from Indonesia, 175 (15.3) from Malaysia and 60 (5.2%) from Vietnam. The majority of participants were female (65.1%) and married (54.2%), with a mean age of 31.7 (s.d. = 7.8) years. Notably, the study cohort from India was younger (27.7 years, s.d. = 5.7) compared with the general study population. The majority of participants were medically trained (755, 65.8%), of which 58.8% (444) were nurses and the remaining 41.2% (311) physicians. Of the non-medically trained personnel (*n* = 391), most were clerical staff (167, 14.6%) (Table 1).

**Table 1:** Baseline characteristics of the study participants (*n* = 1146)

Characteristics	Overall (*n* = 1146)	Singapore (*n* = 277)	India (*n* = 384)	Malaysia (*n* = 175)	Vietnam (*n* = 60)	Indonesia (*n* = 250)
Female gender, *n* (%)	746 (65.1)	193 (69.7)	251 (65.4)	118 (67.4)	44 (73.3)	140 (56.0)
Age, mean (s.d.)	31.7 (7.8)	35.0 (9.2)	27.7 (5.7)	32.4 (6.5)	34.7 (9.6)	33.2 (7.0)
Ethnicity, *n* (%)						
Chinese	154 (13.4)	136 (49.1)	0 (0.0)	12 (6.9)	0 (0.0)	6 (2.4)
Indian	435 (38.0)	46 (16.6)	383 (99.7)	6 (3.4)	0 (0.0)	0 (0.0)
Malay	211 (18.4)	29 (10.5)	1 (0.3)	58 (33.1)	0 (0.0)	123 (49.2)
Eurasian	3 (0.3)	2 (0.7)	0 (0.0)	0 (0.0)	0 (0.0)	1 (0.4)
White	28 (2.4)	25 (9.0)	0 (0.0)	0 (0.0)	3 (5.0)	0 (0.0)
Other	315 (27.5)	39 (14.1)	0 (0.0)	99 (56.6)	57 (95.0)	120 (48.0)
Marital status						
Single	504 (44.0)	122 (44.0)	237 (61.7)	66 (37.7)	24 (40.0)	55 (22.0)
Married	621 (54.2)	145 (52.4)	146 (38.0)	103 (58.9)	35 (58.3)	192 (76.8)
Divorced/separated/widowed	21 (1.8)	10 (3.6)	1 (0.3)	6 (3.4)	1 (1.7)	3 (1.2)
Occupation						
Physician	311 (27.1)	77 (27.8)	84 (21.9)	62 (35.4)	28 (46.7)	60 (24)
Nurse	444 (38.7)	99 (35.7)	167 (43.5)	94 (53.7)	20 (33.3)	64 (25.6)
Technician	70 (6.1)	21 (7.6)	42 (10.9)	3 (1.7)	4 (6.7)	0 (0.0)
Clerical staff/executive	167 (14.6)	14 (5.1)	40 (10.4)	3 (1.7)	5 (8.3)	105 (42.0)
Administrator	48 (4.2)	16 (5.8)	24 (6.3)	6 (3.4)	2 (3.3)	0 (0.0)
Maintenance worker	33 (2.9)	9 (3.2)	21 (5.5)	2 (1.1)	1 (1.7)	0 (0.0)
Allied professional	73 (6.4)	41 (14.8)	6 (1.6)	5 (2.9)	0 (0.0)	21 (8.4)
Prior medical conditions						
Hypertension	65 (5.7)	15 (5.4)	10 (2.6)	19 (10.9)	5 (8.3)	16 (6.4)
Dyslipidaemia	43 (3.8)	7 (2.5)	6 (1.6)	7 (4.0)	5 (8.3)	18 (7.2)
Diabetes mellitus	23 (2.0)	6 (2.2)	3 (0.8)	4 (2.3)	5 (8.3)	5 (2.0)
Asthma	60 (5.2)	15 (5.4)	6 (1.6)	13 (7.4)	1 (1.7)	25 (10)
Eczema	43 (3.8)	16 (5.8)	0 (0)	5 (2.9)	3 (5)	19 (7.6)
Migraine	109 (9.5)	26 (9.4)	20 (5.2)	17 (9.7)	5 (8.3)	41 (16.4)
Ischaemic heart disease	6 (0.5)	0 (0.0)	1 (0.3)	0 (0.0)	0 (0.0)	5 (2.0)
Stroke	3 (0.3)	0 (0.0)	0 (0.0)	1 (0.6)	0 (0.0)	2 (0.8)
Others	32 (2.8)	16 (5.8)	2 (0.5)	0 (0.0)	3 (5)	11 (4.4)
Smoking	37 (3.2)	17 (6.1)	11 (2.9)	2 (1.1)	1 (1.7)	6 (2.4)
Physical symptoms						
Throat pain	289 (25.2)	58 (20.9)	143 (37.2)	26 (14.9)	10 (16.7)	52 (20.8)
Nausea	88 (7.7)	11 (4.0)	40 (10.4)	7 (4.0)	0	30 (12.0)
Anxiety	367 (32.0)	80 (28.9)	121 (31.5)	33 (18.9)	28 (46.7)	105 (42.0)
Insomnia	302 (26.4)	82 (29.6)	106 (27.6)	43 (24.6)	22 (36.7)	49 (19.6)
Poor appetite	218 (19.0)	25 (9.0)	122 (31.8)	20 (11.4)	12 (20.0)	39 (15.6)
Lethargy	415 (36.2)	96 (34.7)	132 (34.4)	50 (28.6)	11 (18.3)	126 (50.4)
Watery eyes	48 (4.2)	16 (5.8)	8 (2.1)	8 (4.6)	3 (5.0)	13 (5.2)
Pruritus	99 (8.6)	23 (8.3)	15 (3.9)	10 (5.7)	5 (8.3)	46 (18.4)
Myalgia	328 (28.6)	74 (26.7)	136 (35.4)	43 (24.6)	8 (13.3)	67 (26.8)
Rash	66 (5.8)	15 (5.4)	20 (5.2)	10 (5.7)	1 (1.7)	20 (8.0)
Coryza	137 (12.0)	74 (26.7)	136 (35.4)	43 (24.6)	8 (13.3)	67 (26.8)
Breathlessness	83 (7.2)	23 (8.3)	31 (8.1)	11 (6.3)	1 (1.7)	17 (6.8)
Cough	166 (14.5)	52 (18.8)	10 (2.6)	31 (17.7)	7 (11.7)	66 (26.4)
Sputum	109 (9.5)	38 (13.7)	13 (3.4)	12 (6.9)	7 (11.7)	39 (15.6)
Headache	384 (33.5)	96 (34.7)	158 (41.1)	50 (28.6)	18 (30.0)	62 (24.8)
Neck stiffness	157 (13.7)	53 (19.1)	18 (4.7)	29 (16.6)	6 (10.0)	51 (20.4)

**Fig. 1:**
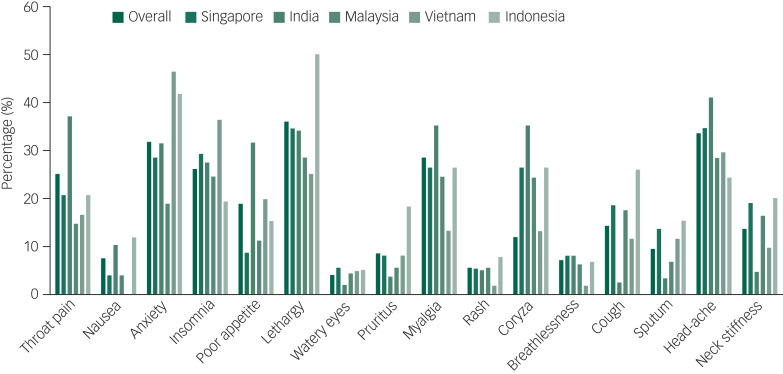
Prevalence of physical symptoms experienced by healthcare workers by country during the COVID-19 pandemic.

The commonest reported symptoms were lethargy (415, 36.2%) and headache (384, 33.5%) (Fig. 1). Almost one-third (375, 32.7%) of participants did not have any symptoms in the 1 month prior to survey administration. On the other hand, 124 (10.8%) reported one symptom, 119 (10.4%) reported two symptoms and 528 (46.1%) reported three or more symptoms. Most of the reported symptoms were rated as mild in severity.

### Comparison of adverse psychological outcomes between countries

**Fig. 2:**
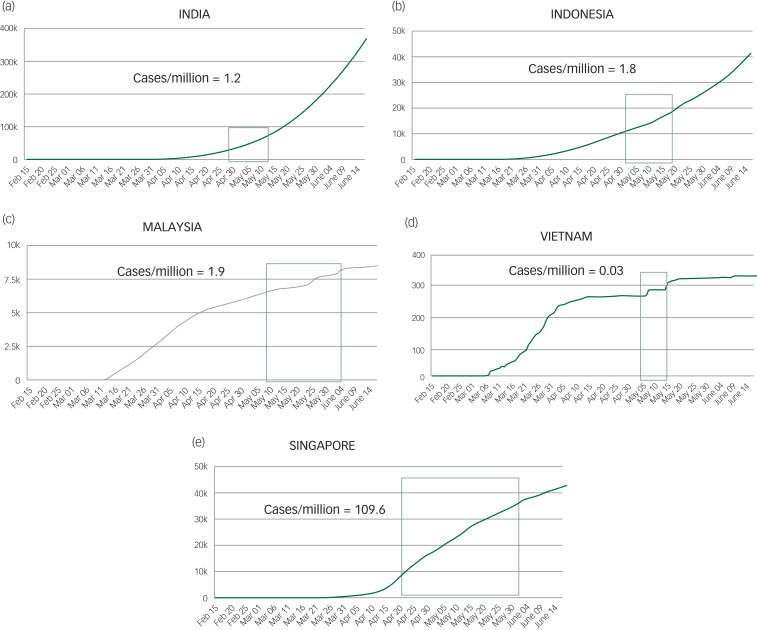
The longitudinal trajectory of confirmed cases of COVID-19 in the participating countries. Total number of confirmed COVID-19 cases per 1 million population for each country is also represented. (a) India; (b) Indonesia; (c) Malaysia; (d) Vietnam; (e) Singapore. The denoted section of the trend line (green box) represents the study period in each country.

**Fig. 3:**
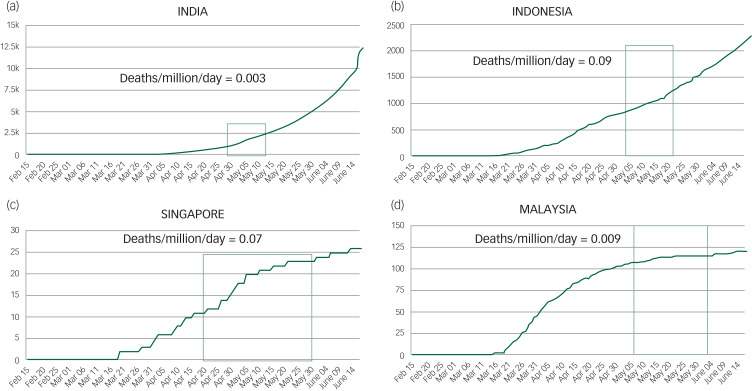
The longitudinal trajectory of confirmed deaths because of COVID-19 in the participating countries. Total number of confirmed deaths because of COVID-19 per 1 million population for each country is also represented. (a) India; (b) Indonesia; (c) Singapore; (d) Malaysia. The denoted section of the trend line (green box) signifies the study period of each country.

During each of the countries’ recruitment period, the total confirmed cases per day per 1 million population and deaths per day/per 1 million population were collated (Figs. 2 and 3). Singapore had the highest number of cases (109.6 per day/1 million population) followed by Malaysia (1.9 cases per day/1 million population), Indonesia (1.8 cases per day/1 million population), India (1.2 cases per day/1 million population) and Vietnam (0.03 cases per day/1 million population).

During the COVID-19 pandemic in various Asia-Pacific countries, 51 (4.5%) of participants of our overall study cohort of healthcare workers screened positive for depression, 60 (5.2%) for anxiety, 12 (1.0%) for stress, and 91 (7.9%) for PTSD related to COVID-19. The overall mean DASS-21 depression, anxiety and stress scores were 1.89 (s.d. = 3.10), 1.95 (s.d. = 2.75), and 2.75 (s.d. = 3.50), respectively. The mean total IES-R score was 8.92 (s.d. = 9.76), while the mean scores for IES-R subsets for intrusion, avoidance and hyperarousal were 0.42 (s.d. = 0.47), 0.42 (s.d. = 0.52) and 0.40 (s.d. = 0.47), respectively.

**Fig. 4:**
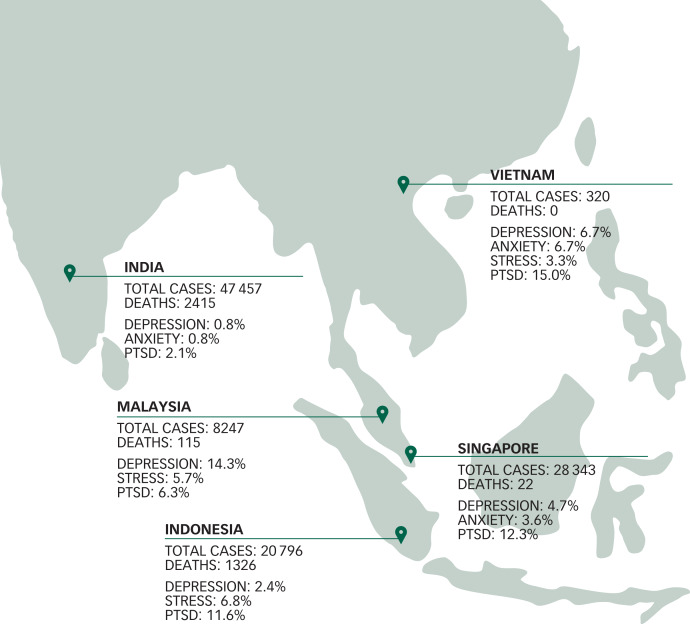
The total number of confirmed COVID-19 cases and deaths at the end of the country's study period, with the prevalence of adverse psychological outcomes among healthcare workers in each country (*n* = 1146). The Depression Anxiety Stress Scales (DASS-21) was used, in which DASS-21 cut-off scores of >9, >7 and >14 indicate a positive screen of depression, anxiety and stress, respectively. Post-traumatic stress disorder was screened using the Impact of Events Scale-Revised (IES-R) score, where a total IES-R cut-off score of 24 is used to classify post-traumatic stress disorder as a clinical concern. India and Singapore did not have any healthcare workers screen positive for stress. PTSD, post-traumatic stress disorder.

In the individual country subgroup analysis, India displayed the lowest prevalence of adverse psychological outcomes. India had the lowest prevalence of depression (0.8%), followed by Indonesia (2.4%) Singapore (4.7%), Vietnam (6.7%) and Malaysia (14.3%). Again, India had the lowest prevalence (0.8%) of anxiety, followed by Singapore (3.6%), Vietnam (6.7%) Indonesia (6.8%) and Malaysia (14.9%). Interestingly, the prevalence of stress was low in most countries, with figures of 5.7% in Indonesia and 3.3% in Vietnam, but none screened positive in Singapore and India (Fig. 4). Overall, there were significant differences in the prevalence of adverse psychological outcomes between the five countries. Using India as the reference group, overall results are displayed in Table 2.

**Table 2:** Comparison of the prevalence of depression, anxiety and PTSD in healthcare workers in the five countries during the COVID-19 pandemic (*n* = 1146)

Countries	Depression			Anxiety			PTSD		
	*n* (%)	OR (95% CI)	*P*	*n* (%)	OR (95% CI)	*P*	*n* (%)	OR (95% CI)	*P*
Indonesia	6 (2.4)	3.123 (0.774–12.603)	0.110	17 (6.8)	9.266 (2.686–31.961)	**<0.001**	29 (11.6)	6.167 (2.771–13.728)	**<0.001**
Singapore	13 (4.7)	6.254 (1.765–22.161)	**0.005**	10 (3.6)	4.757 (1.297–17.447)	**0.019**	34 (12.3)	6.576 (2.994–14.445)	**<0.001**
Malaysia	25 (14.3)	21.167 (6.297–71.150)	**<0.001**	26 (14.9)	22.161 (6.608–74.317)	**<0.001**	11 (6.3)	3.152 (1.245–7.982)	**0.015**
Vietnam	4 (6.7)	9.071 (1.978–41.601)	**0.005**	4 (6.7)	9.071 (1.978–41.601)	**0.005**	9 (15.0)	8.294 (3.063–22.462)	**<0.001**
India	3 (0.8)	Reference	Reference	3 (0.8)	Reference	Reference	8 (2.1)	Reference	Reference

Results in bold are significant.

**Fig. 5:**
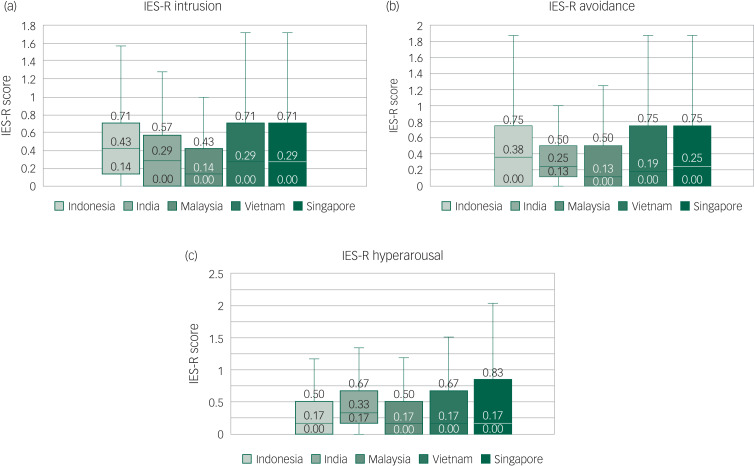
The mean Impact of Events Scale Revised (ES-R) scores for each country, categorised into its subscales of (a) intrusion, (b) avoidance and (c) hyperarousal (*n* = 1146).

The IES-R system was used to evaluate the extent of psychological distress among healthcare workers. Healthcare workers from India displayed the lowest prevalence of PTSD related to COVID-19 (2.1%), followed by Malaysia (6.3%), Indonesia (11.6%), Singapore (12.3%) and Vietnam (15.0%). The comparison of IES-R subsets of intrusion, avoidance and hyperarousal between the five countries are displayed in Fig. 5.

### Predictors of adverse psychological outcomes

Multivariate analysis of healthcare workers was performed to assess independent predictors of anxiety for each country, and the variables of the model included gender (female), non-medically trained personnel, presence of prior medical conditions (past medical history), presence of symptoms and age. The multivariate analysis model for depression showed the presence of physical symptoms as the independent predictor of depression in the Malaysian (odds ratio (OR) = 5.673, 95% CI 1.780–18.075, *P* = 0.003) and Indonesian cohort (OR = 1.241, 95% CI 1.010–1.526, *P* = 0.040) (Table 3).

**Table 3:** Multivariate analysis of healthcare workers in each country with depression during the COVID-19 pandemic (*n* = 1146)

Variables	Overall (*n* = 1146)		Singapore (*n* = 277)		India (*n* = 384)		Malaysia (*n* = 175)		Vietnam (*n* = 60)		Indonesia (*n* = 250)	
	OR (95% CI)	*P*	OR (95% CI)	*P*	OR (95% CI)	*P*	OR (95% CI)	*P*	OR (95% CI)	*P*	OR (95% CI)	*P*
Gender (female)	0.755 (0.515–1.105)	0.148	0.221 (0.027–1.769)	0.155	0.452 (0.027–7.699)	0.583	2.347 (0.912–6.038)	0.077	0.349 (0.011–11.470)	0.555	0.365 (0.039–3.387)	0.376
Non-medically trained personnel	1.832 (1.267–2.653)	0.001	1.186 (0.339–4.153)	0.790	1.188 (0.087–16.234)	0.897	0.272 (0.033–2.271)	0.229	0.662 (0.309–1.415)	0.287	5.399 (0.569–51.271)	0.142
Presence of prior medical conditions	1.346 (0.886–2.045)	0.164	1.310 (0.350–4.901)	0.688	1.739 (0.088–34.381)	0.716	0.303 (0.076–1.210)	0.091	0.284 (0.012–6.582)	0.432	0.670 (0.098–4.580)	0.683
Presence of symptoms	3.185 (1.916–5.291)	<0.001	6.017 (0.740–48.919)	0.093	1.332 (0.860–2.062)	0.199	5.673 (1.780–18.075)	**0.003**	1.127 (0.085–15.029)	0.928	1.241 (1.010–1.526)	**0.040**
Age	1.005 (0.983–1.028)	0.657	0.988 (0.923–1.057)	0.719	1.090 (0.948–1.254)	0.228	1.002 (0.920–1.092)	0.959	1.173 (0.949–1.451)	0.140	0.936 (0.797–1.100)	0.422

Results in bold are significant.

The study found that being a non-medically trained healthcare personnel was an independent predictor for anxiety in the Indonesian cohort (OR = 4.908, 95% CI 1.282–18.789, *P* = 0.020) after adjusting for confounders. In the Singapore cohort, the presence of prior medical conditions was an independent predictor for anxiety (OR = 5.828, 95% CI 1.397–24.308, *P* = 0.016) (Table 4).

**Table 4:** Multivariate analysis of healthcare workers in each country with anxiety during the COVID-19 pandemic (*n* = 1146)

Variables	Overall (*n* = 1146)		Singapore (*n* = 277)		India (*n* = 384)		Malaysia (*n* = 175)		Vietnam (*n* = 60)		Indonesia (*n* = 250)	
	OR (95% CI)	*P*	OR (95% CI)	*P*	OR (95% CI)	*P*	OR (95% CI)	*P*	OR (95% CI)	*P*	OR (95% CI)	*P*
Gender (female)	0.904 (0.684–1.193)	0.477	0.594 (0.117–3.016)	0.530	1.155 (0.069–19.390)	0.920	1.537 (0.634–3.729)	0.342	0.324 (0.012–8.663)	0.502	0.419 (0.130–1.351)	0.145
Non-medically trained personnel	1.173 (0.880–1.563)	0.277	1.587 (0.420–6.000)	0.496	1.211 (0.074–19.870)	0.893	0.775 (0.159–3.780)	0.752	0.662 (0.309–1.415)	0.287	4.908 (1.282–18.789)	**0.020**
Presence of prior medical conditions	1.126 (0.814–1.558)	0.474	5.828 (1.397–24.308)	**0.016**	5.774 (0.430–77.551)	0.186	1.432 (0.483–4.249)	0.517	0.259 (0.009–7.576)	0.433	0.906 (0.304–2.693)	0.858
Presence of symptoms	1.408 (1.052–1.890)	0.022	4.502 (0.544–37.257)	0.163	0.693 (0.046–10.503)	0.791	1.735 (0.685–4.393)	0.245	0.808 (0.050–12.946)	0.880	4.660 (0.587–37.014)	0.145
Age	0.996 (0.978–1.013)	0.627	1.001 (0.938–1.069)	0.966	0.917 (0.693–1.214)	0.546	0.903 (0.816–1.000)	0.051	1.120 (0.941–1.332)	0.201	0.999 (0.931–1.071)	0.967

Results in bold are significant.

In the multivariate analysis, the independent predictors of PTSD related to COVID-19 were non-medically trained personnel in the Singaporean (OR = 2.729, 95% CI 1.150–6.472, *P* = 0.023) and Indonesian cohort (OR = 2.443, 95% CI 1.004–5.942, *P* = 0.049). Additionally, the presence of prior medical conditions (OR = 2.425, 95% CI 1.014–5.802, *P* = 0.046) and presence of symptoms (OR = 6.692, 95% CI 1.517–29.521, *P* = 0.012) were independent predictors of PTSD in the Singaporean cohort (Table 5).

**Table 5:** Multivariate analysis of healthcare workers in each country with post-traumatic stress disorder during the COVID-19 pandemic (*n* = 1146)

Variables	Overall (*n* = 1146)		Singapore (*n* = 277)		India (*n* = 384)		Malaysia (*n* = 175)		Vietnam (*n* = 60)		Indonesia (*n* = 250)	
	OR (95% CI)	*P*	OR (95% CI)	*P*	OR (95% CI)	*P*	OR (95% CI)	*P*	OR (95% CI)	*P*	OR (95% CI)	*P*
Gender (female)	1.113 (0.692–1.791)	0.658	1.261 (0.511–3.115)	0.615	0.514 (0.059–4.466)	0.546	1.854 (0.520–6.613)	0.341	1.686 (0.302–9.426)	0.552	0.940 (0.421–2.097)	0.880
Non-medically trained personnel	1.418 (0.887–2.273)	0.145	2.729 (1.150–6.472)	**0.023**	0.234 (0.026–2.115)	0.196	1.005 (0.112–8.994)	0.996	2.420 (0.244–23.966)	0.450	2.443 (1.004–5.942)	**0.049**
Presence of prior medical conditions	1.754 (1.059–2.907)	0.029	2.425 (1.014–5.802)	**0.046**	1.129 (0.124–10.264)	0.914	1.422 (0.318–6.348)	0.645	0.357 (0.033–3.822)	0.394	1.433 (0.628–3.273)	0.393
Presence of symptoms	2.387 (1.289–4.425)	0.006	6.692 (1.517–29.521)	**0.012**	1.225 (0.273–5.500)	0.791	3.578 (0.722–17.735)	0.118	1.198 (0.227–6.328)	0.832	2.383 (0.676–8.400)	0.177
Age	1.018 (0.991–1.045)	0.204	1.012 (0.969–1.056)	0.602	0.992 (0.867–1.135)	0.907	0.947 (0.835–1.075)	0.400	1.034 (0.920–1.162)	0.574	0.975 (0.917–1.038)	0.433

Results in bold are significant.

## Discussion

### Main findings

To our knowledge, this is the first multicentre study that has examined the prevalence of psychological outcomes among healthcare workers during the evolution of the COVID-19 pandemic in the Asia-Pacific region. Our study demonstrated the discrepancy between the volume of confirmed COVID-19 cases per day/1 million population and the prevalence of adverse psychological outcomes in the participating countries. Despite having the lowest volume of cases per day/1 million population, Vietnam displayed a higher prevalence of PTSD related to COVID-19 among healthcare workers compared with India. In contrast, Singapore reported the highest number of cases per day/1 million population, but had a lower prevalence of depression and anxiety among its healthcare workers, when compared with the Malaysian cohort. Our study highlights that all healthcare workers were vulnerable to psychological adversity regardless of the volume of confirmed COVID-19 cases. In fact, it would be crucial to focus efforts on addressing the independent predictors of psychological adversity, such as healthcare workers with the presence of physical symptoms, presence of prior medical conditions and those who are not medically trained.

### Comparison with findings from other studies

During the early stages of the COVID-19 pandemic, Wang et al investigated the psychological impact of the COVID-19 pandemic on the general public in China using the DASS-21 scale, and found that 16.5%, 28.8% and 8.1% of its respondents reported moderate-severe depressive, anxiety and stress levels, respectively.^12^ However, given the differences in sampling approach, measurement and methodology, it poses a challenge for direct comparison with our present study cohorts’ prevalence of depression (1.5%), anxiety (3.0%) and stress (0.09%). We speculate that the prevalence of adverse psychological outcomes may improve across the COVID-19 pandemic trajectory, as there will have been an additional amount of time and effort in countries in building their readiness through resource allocation, emergency implementation of drastic infection control measures and mental preparedness.^13,14^

Our earlier study reported depression rates among non-medical and medical healthcare personnel to be 10.3% and 8.1% respectively, as compared with the overall 4.7% depression rate displayed in the present study.^5^ This trend was similar for the rates of anxiety in the early phases of the pandemic (20.7% in non-medical and 10.8% in medical healthcare workers, versus the overall 3.6% in the present study).^5^ It is important to note that it is difficult to make accurate longitudinal conclusions when comparing the two studies because of the different population cohorts. Nevertheless, there is a trend towards better psychological preparedness and resilience for impending widespread transmission when the spread of the pandemic is already known, as compared with the uncertainty during the earlier stages of the pandemic.^15^

### Impact of differing strategies in different countries

Each of the participating countries has adopted strategies to curb the spread of COVID-19 and ensuring confidence in its healthcare workers through preventive strategies. The world witnessed the largest COVID-19 national lockdown in India, which led to the desired effect of flattening the epidemic curve. India's population of 1.3 billion with its widening socioeconomic disparities pose unique challenges in the pandemic. Moreover, India's young general population (65% of the population are less than 35 years of age) may serve as a determining factor for psychological resilience as evident by the lower levels of adverse psychological outcomes in this study.^16^ This characteristic is reflected in the present study's Indian cohort with a lower mean age with fewer prior medical conditions as compared with their counterparts.

The most effective lockdown was seen in Vietnam. The country tightened its border immediately after the first death in Wuhan. Vietnam's approach was never based on mass testing, which may possibly account for its strikingly low number of confirmed cases. Its stringent infectious control has been backed up by the country's military and public security forces.^17^ Despite their low levels of detected cases, an interesting finding of our study is Vietnam's high prevalence of PTSD, and relative higher rates of anxiety and depression as compared with their India and Singapore counterparts.

Singapore adopted a multipronged aggressive surveillance strategy among various patient groups (for example all patients with pneumonia) that allowed clinician's discretion to order a test based on clinical suspicion, even if the case definition was not met.^18^ This strategy led to an increase in detected COVID-19 cases, many of which may not have been detected if the case definition was strictly followed. Although, it reported a high volume of confirmed cases, most were asymptomatic or mildly symptomatic.^18^ Perhaps, this accounted for the lower prevalence of anxiety, depression and PTSD related to COVID-19 among healthcare workers.

Similarly, Malaysia adopted an aggressive testing strategy, in which its tests per million capita far exceed its Association of South-East Asian Nations counterparts, resulting in higher reported confirmed cases but of milder severity and lower mortality.^19^ However, despite its lower rates of confirmed cases as compared with Singapore, we found a higher prevalence of depression, anxiety and stress among Malaysian healthcare workers.

Indonesia's declaration of a public health emergency on 21 March led to large-scale social distancing and movement restriction.^20^ Despite its higher death rates per 1 million population, the prevalence of depression and anxiety remain comparable with its counterparts. Therefore, there is no clear correlation between the volume of confirmed cases per day per million capita and the prevalence of adverse psychological outcomes. Perhaps the risk of psychological distress among healthcare workers goes beyond the country's COVID-19 disease burden, and may also be contributed to by other societal and cultural factors specific to different nations.

### Differences between medical and non-medical healthcare workers

Our study demonstrated that non-medically trained healthcare workers were at higher risk of adverse psychological outcomes as compared with their medically trained counterparts. This is in agreement with our recent study on healthcare workers during the start of the pandemic in Singapore, as well as a recent study in China that demonstrated that front-line nurses had significantly decreased vicarious traumatisation scores in comparison with non-front-line nurses and the general public during the COVID-19 pandemic.^5,21^ This difference between medical and non-medical healthcare workers may be attributed to the relative lack of accessibility to first-hand medical information on the pandemic and less formal training and confidence in infectious control measures.^5^

### Role of physical symptoms

Our current findings demonstrate that the presence of physical symptoms was an independent predictor of adverse psychological outcomes. This is in line with our previous study that reported this significant association during the earlier stages of the pandemic.^22^ We postulated a bidirectional association between physical symptoms and psychological stress, in which somatic symptoms may represent a way of communicating emotions.^23^ However, healthcare workers displaying physical symptoms may face a sense of fear, stigmatisation and ostracism from co-workers, which may exacerbate the psychological pain.^24–26^

### Role of prior medical conditions

We found that healthcare workers with prior medical conditions were at risk of adverse psychological outcomes. Studies have shown that vulnerable groups, namely those who are immunocompromised, with underlying medical conditions are at higher risk of life-threatening illness from COVID-19.^27,28^ The knowledge of this increased risk may have worked as an additional stressor for our healthcare workers with pre-existing illnesses. Perhaps more efforts can be made to address the concerns of this vulnerable group of healthcare workers through ensuring that their exposure to COVID-19 patients are adjusted accordingly to their health risk status.

### Implications

Our present findings demonstrate that regardless of the volume of cases or deaths, healthcare workers from all countries are vulnerable to psychological distress from the COVID-19 outbreak. Therefore, as the pandemic reaches its peak, it calls for urgent clinical and policy strategies for identifying healthcare workers at risk, i.e. those who are not medically trained, those with physical symptoms and prior medical conditions. Passive psychoeducation through educational pamphlets, emails or website can be relatively easy and inexpensive to implement, and may serve as a readily available resource for those experiencing psychological distress. Education on the natural history of the virus, and the appropriate use of infection control measures, especially for the non-medically trained hospital workers, may be helpful. A meta-analysis has shown that brief passive psychoeducational interventions targeting high-risk groups can be effective, although the extent of benefit remains unknown.^29^ Early psychological interventions in the form of cognitive–behavioural therapy, have also been proven to reduce psychological distress.^30^ Therefore, improved accessibility to formal psychological support for healthcare workers (in the form of counselling, internet-based cognitive–behavioural therapy) is of paramount importance.^5^ These dedicated interventions will help allay the fear of COVID-19 transmission between colleagues and improve the confidence of our healthcare workers.^12,22^

### Limitations

We acknowledge certain limitations of the study. First, it is cross-sectional in nature, which does not allow us to assess the causality of the different psychological outcomes. We also did not examine the prevalence of psychological symptoms before COVID-19, and did not examine the participants longitudinally for progression or improvement in their symptoms. Second, questionnaires were self-administered because of the strict infection control measures in all participating institutions. Hence, the information obtained could not be verified by a medical professional. Furthermore, all surveys were administered in English. Although English had been the primary language of communication in all the hospitals in which the survey had been administered, varying levels of proficiency with the English language may also have contributed to bias and inaccuracy in the survey findings.

In terms of sampling, larger tertiary centres within the countries were studied. The findings thus may not be generalisable and reflective of smaller regional or rural centres. Although the response rate was greater than 88%, participants with greater mental health concerns may be more likely to respond to the study, which may slightly overestimate the prevalence of mental health disorders. A larger sample size that is more representative of healthcare workers across the study region would be needed to estimate the true prevalence of mental disorders in this population. Follow-up studies are required to evaluate the progression of the psychological impact on healthcare workers during the aftermath of the COVID-19 pandemic, as there have been concerns regarding the ‘rebound effect’ where high-risk healthcare workers may experience various neuropsychiatric manifestations once the imminent threat of the infectious disease subsides, along with the protective positive attitude of collegiality and bravery.^31^

In conclusion, we reported a varied, albeit high, prevalence of psychological distress in healthcare workers regardless of the individual countries’ burden of confirmed COVID-19 cases and deaths, as well as the different infectious control strategies adopted by the various countries. Our study suggested that vulnerable groups of healthcare workers may include those who are not medically trained, and those with physical symptoms and prior medical conditions. Targeted psychological interventions may be beneficial for this group of healthcare workers.

## Data Availability

The data that support the findings of this study are available on request from the corresponding author, B.Y.Q.T.
